# Effect of a Combination of Magnesium, B Vitamins, Rhodiola, and Green Tea (L-Theanine) on Chronically Stressed Healthy Individuals—A Randomized, Placebo-Controlled Study

**DOI:** 10.3390/nu14091863

**Published:** 2022-04-29

**Authors:** Lionel Noah, Veronique Morel, Claire Bertin, Etienne Pouteau, Nicolas Macian, Christian Dualé, Bruno Pereira, Gisèle Pickering

**Affiliations:** 1 Sanofi, 82, Avenue Raspail, 94250 Gentilly, France; claire.bertin@sanofi.com (C.B.); etienne.pouteau@sanofi.com (E.P.); 2 CIC INSERM 1405/Plateforme d’Investigation Clinique CHU Gabriel Montpied, 58 Rue Montalembert, CEDEX 1, 63000 Clermont-Ferrand, France; v_morel@chu-clermontferrand.fr (V.M.); nmacian@chu-clermontferrand.fr (N.M.); cduale@chu-clermontferrand.fr (C.D.); bpereira@chu-clermontferrand.fr (B.P.); gisele.pickering@uca.fr (G.P.)

**Keywords:** chronic stress, Depression Anxiety Stress Scale-42 questionnaire, green tea, L-theanine, magnesium, Pittsburgh Sleep Quality Index, pain, vitamins, rhodiola, randomized clinical trial

## Abstract

The effect of a combination of magnesium, vitamins B6, B9, B12, rhodiola and green tea/L-theanine (Mg-Teadiola) on stress was evaluated in chronically stressed, otherwise healthy individuals. Effects on stress-related quality-of-life parameters (sleep and perception of pain) were also explored. Adults with stress for ≥1 month, scoring ≥14 points on the Depression Anxiety Stress Scale (DASS)-42 questionnaire, were randomized (1:1) to receive oral Mg-Teadiola (*n* = 49) or a placebo (*n* = 51), for 28 days, with a follow-up assessment on Day 56 (NCT04391452). The primary endpoint was the change in the DASS-42 stress score from baseline to Day 28 with Mg-Teadiola versus placebo. The DASS-42 stress scores significantly decreased from baseline to Day 28 with Mg-Teadiola versus placebo (effect size, 0.29; 95% CI [0.01, 0.57]; *p* = 0.04). Similar reductions were observed on Day 14 (*p* = 0.006) and Day 56 (*p* = 0.02). A significant reduction in sensitivity to cold pain (*p* = 0.01) and a trend for lower sensitivity to warm pain was observed (*p* = 0.06) on Day 28. Improvements in daytime dysfunction due to sleepiness (Pittsburgh Sleep Quality Index-7 component score) were reported on Day 28, and were significant on Day 56 (*p* < 0.001). Mg-Teadiola is effective in managing stress in otherwise healthy individuals. Its beneficial effects on sleep and pain perception need further investigation.

## 1. Introduction

Stress is a common health problem with a global prevalence of 29.6% in the general population and 44.9% among healthcare professionals [1,2]. Chronic stress can have devastating effects on physical and mental health, and results in comorbidities such as anxiety and pain—thus impacting quality of life (QoL) [3]. 

Anxiolytics, sedatives, anti-depressants, and beta-blockers are often prescribed for relieving stress-related symptoms [4,5]. Considering the adverse effects of existing drug therapies, there is a need to identify relevant options that are better adapted to the needs of healthy populations for the management of stress.

There has been extensive research into herbal extracts and mineral supplements to explore their benefits in stress and anxiety. Studies have demonstrated that magnesium (Mg), green tea and rhodiola extracts, and vitamin B6 possess stress- and anxiety-relieving effects [6,7,8,9]. Multivitamins containing vitamin B or high-dose vitamin B complex have been shown to reduce stress in healthy volunteers [10,11]. Recently, a potential protective effect of a combination of Mg, vitamins B6, B9 and B12, and rhodiola and green tea (referred to as Mg-Teadiola in this article) on acute induced social stress in healthy individuals has been reported [12,13]. However, there is a limited number of robust, placebo-controlled, randomized clinical trials evaluating the effect of dietary supplements in healthy individuals who report stress.

In the present clinical study, we evaluated the effect of the dietary supplement Mg-Teadiola versus a placebo in stressed, healthy individuals scoring at least 14 points on a clinically validated, self-reported measure—the Depression Anxiety Stress Scale (DASS) [14]. We also explored the effects of Mg-Teadiola on a variety of QoL parameters related to stress: anxiety, depression [15,16], quality of sleep [17,18], and perception of pain [19,20].

## 2. Materials and Methods

### 2.1. Study Design

This was a 28-day, randomized, single-blinded, placebo-controlled, parallel-group study, conducted from July 2020 to July 2021 at the Centre for Clinical Pharmacology, Clermont-Ferrand, France.

**Figure 1:**
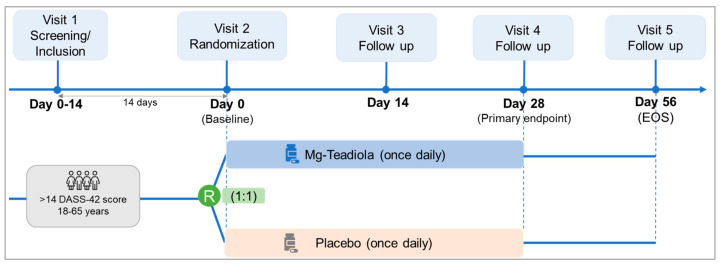
Study design. DASS, Depression Anxiety Stress Scale; EOS, end of study; Mg-Teadiola, combination of 150 mg magnesium, 0.7 mg vitamin B6, 0.1 mg vitamin B9, and 1.25 µg vitamin B12, +222 mg of rhodiola extract and 125 mg of green tea extract including 50 mg of L-theanine; R, randomization.

The study included five visits: a screening visit (Visit 1, 14 days before Visit 2), a randomization visit (Visit 2, baseline, Day 0), two treatment follow-up visits (Visits 3 and 4 on Days 14 and 28, respectively) and one end-of-study follow-up visit (Visit 5 on Day 56, Figure 1).

The ethics committee (the Committee for the Protection of Persons [CPP]: Sud EST II, France) approved the study protocol on 11 March 2020. The study was conducted according to the Good Clinical Practice guidelines and the World Medical Association Declaration of Helsinki (Tokyo 2004, revised). Participants provided informed consent before participating in this study. This study is registered at the National Agency for the Safety of Medicines and Healthcare Products French register (2020-A00040-39, approval date: 11 March 2020) and clinicaltrials.gov (NCT04391452).

### 2.2. Participants

Participants were screened from the volunteer database of the Clinical Pharmacology Center/Clinical Investigation Center Inserm 1405 in Clermont-Ferrand, France.

Adults (18 to 65 years of age), of either sex, reporting stress for at least one month, who scored greater than or equal to 14 on the DASS-42 stress questionnaire, who were willing to not introduce any new treatment or diet at the time of inclusion, and who were not receiving any treatment (including analgesics and anti-inflammatory drugs) at least 7 days before enrollment were eligible to participate in this study.

The key exclusion criteria were contraindication or hypersensitivity to Mg administration, magnesemia greater than 1.07 mmol/L, and severe renal failure with creatinine clearance less than 60 mL/min. Individuals treated with antibiotics at least 3 months before inclusion or receiving treatment or dietary supplements containing Mg or herbal extracts at the time of inclusion were not included. Details on the exclusion criteria are provided in supplementary material: exclusion criteria.

### 2.3. Randomization, Masking, and Treatment

The allocation of treatments followed a predefined randomization plan and was performed by an individual who did not contribute to the study protocol. To avoid bias, a randomization list was placed in a sealed envelope and was blinded from the participants, investigator and other personnel involved in this study. All prespecified analyses were performed before treatment groups were unmasked. Eligible participants were randomized (1:1) to orally receive either Mg-Teadiola or placebo once a day in the morning for 28 days. The randomization code was computer-generated through randomly permuted blocks. Within each block, the number of participants allocated to each of the two treatment arms was equal. Mg-Teadiola consisted of a tablet (150 mg of Mg, 0.7 mg of vitamin B6, 0.1 mg of vitamin B9, and 1.25 µg of vitamin B12, and Teadiola^®^ (222 mg of rhodiola extract and 125 mg of green tea extract including 50 mg of L-theanine); Sanofi-Aventis Group). This food supplement provided approximately 30–50% of daily reference values of each micronutrient [21]. The placebo consisted of a tablet with excipients.

This study was single-blinded (investigators were completely blinded and participants were partially blinded) as the placebo and Mg-Teadiola tablets lacked structural similarity. The personnel who administered the treatments did not participate in the collection of data for the study.

### 2.4. Study Objectives

The primary objective was to demonstrate the efficacy of Mg-Teadiola in managing stress in healthy individuals.

The secondary (exploratory) objectives were evaluation of analgesic effect of Mg-Teadiola in response to different thermal stimuli; its effect on anxiety, depression, and sleep; and its effect on biological parameters such as the levels of Mg in plasma and urine. Safety data were collected during the study period.

### 2.5. Endpoints and Assessments

The primary endpoint was the change in the DASS-42 stress subscale scores from baseline to Day 28 with Mg-Teadiola compared with placebo. The secondary endpoints included the (i) change in the DASS-42 stress subscale scores from baseline to Days 14 and 56; (ii) change in DASS-42 anxiety scale scores from baseline to Day 14; (iii) change in DASS-42 scale scores of depression from baseline to Day 14; (iv) change in Pain Catastrophizing Scale (PCS) scores from baseline to Days 14 and 28; (v) change in the Pittsburg Sleep Quality Index (PSQI) scores from baseline to Days 14 and 28; (vi) change in levels of Mg in blood and in urine from baseline to Days 14 and 28 following supplementation with Mg-Teadiola compared with placebo; and (vii) frequency of adverse events (AEs).

Participants completed the DASS-42 questionnaire at screening, baseline, and all follow-up visits. The DASS-42 is a 42-item, clinically validated questionnaire for measuring the negative emotional states of depression, anxiety, and stress over the previous week [22,23,24]. Each element was evaluated from zero to three, with zero being “not present” and three being “very frequently present”. The DASS-42 consisted of three subscales: depression, anxiety, and stress scales. Each subscale consisted of 14 questions. Scores were categorized as 0–14 (normal), 15–18 (mild), 19–25 (moderate), 26–33 (severe), or 34+ (extremely severe) [14,25].

The PCS was used to assess catastrophism at baseline and all other visits [26]. It is a 13-item questionnaire for patients to indicate the extent to which they experience different thoughts and emotions during pain. Scores ranged from 0–4 “0: not at all, 4: all the time”. The final score was the sum of the scores for each question.

The PSQI—a 19-item, validated, self-administered questionnaire—was utilized for the assessment of sleep disorders at baseline and all other visits [27]. The questionnaire measured sleep quality during the month before the patient interview. This questionnaire included seven components: subjective sleep quality, sleep latency, sleep duration, usual sleep efficiency, sleep disorders, use of a sleep medication, and poor daytime fitness [27].

A Quantitative Somatosensory Thermotest (QST) [28] with an advanced thermal stimulator thermode connected to a Medoc PATHWAY (Medoc Ltd., Ramat Yishay, Israel) was used to apply thermal stimuli to participants. The stimuli were delivered to the thenar eminence of the dominant hand from a base value of 32 °C and the chosen paradigm was applied. The QST testing included thermal detection and thermal pain thresholds: cold detection and warm detection thresholds and cold pain and warm pain thresholds. From the baseline value of 32 °C, the Medoc PATHWAY delivered an adjustable temperature peak (in cold and heat, depending upon a regular slope of 1 °C) and was controlled by rapid feedback. This device was used to evaluate the thermal detection (when the individual began to feel the thermal change) and pain threshold (when the individual began to feel pain) to heat and cold by calculating the mean of three measures.

Urine samples were collected for 24 h, a day before visits 3 and 4. Urinary Mg levels were analyzed using the Dimension Vista^®^ System Flex^®^ Mg reagent cartridge (Siemens), a modified version of the methyl thymol blue complexometric procedure. Methyl thymol blue formed a blue complex in the presence of Mg and the amount of complex formed was measured using a bichromatic endpoint method to determine the concentration of Mg [29].

### 2.6. Statistical Analyses

#### 2.6.1. Sample Size Calculation

Limited studies have reported clinically relevant differences with DASS stress scores [9,30]. Accordingly, the sample size was estimated based on an expected effect size (ES) of 0.65 with a two-tailed type I error of 5% and a statistical power of 90%. Fifty participants per group were required to highlight such a difference for the primary endpoint.

#### 2.6.2. Statistical Methods

Continuous data were expressed as mean (±standard deviation). The Shapiro–Wilk test was used to analyze the assumption of the Gaussian distribution. Analyses were performed in a modified intention-to-treat set that included participants who had been allocated to a randomized treatment, regardless of whether the treatment had been started or not and had DASS-42 stress scores at baseline and Visit 4 (Day 28). The comparisons between the Mg-Teadiola and placebo groups at Visit 3 to Visit 5 were performed using the analysis of covariance adjusted based on the baseline value of the dependent endpoint [31] and the results were expressed using ES and 95% confidence intervals (CIs). A clinically relevant difference of three points was defined based on the judgement of expert clinicians [9]. When appropriate, a logarithmic transformation of the dependent variables was proposed. No adjustments were made for multiple comparisons. Due to the potential for type I errors due to multiple comparisons, the findings for analyses of secondary analyses were interpreted as exploratory, with no adjustment made for multiple comparisons [32,33].

Factorial discriminant analysis (FDA) was used to determine which parameters could be discriminated between the Mg-Teadiola and placebo groups. FDA is a supervised discrimination method and can be used as a classification technique. This type of supervised discrimination method is used when data sets of well-classified data are available. The selection of parameters to be included in the FDA process was performed according to the univariate results and clinical relevance. The discrimination rules were based on linear combinations of the observed variables that were considered discriminant factors. The selection of factors in FDA can be solved analytically or estimated. The analytical study of the equations involved showed that the discriminant factors minimized the Mahalanobis distance in each group and maximized the distance between groups, providing compact groups that were spread as widely as possible in the space.

Statistical analysis was performed with Stata software (version 15, Stata Corp., College Station, TX, USA) and R software for FDA (package ade4).

## 3. Results

### 3.1. Patient Disposition and Baseline Characteristics

**Figure 2:**
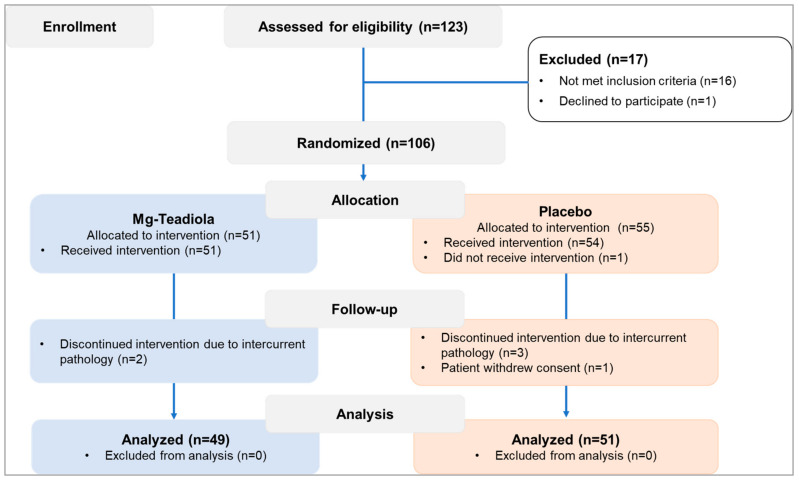
Patient disposition. Mg-Teadiola, combination of 150 mg magnesium, 0.7 mg vitamin B6, 0.1 mg vitamin B9, and 1.25 µg vitamin B12, + 222 mg of rhodiola extract and 125 mg of green tea extract including 50 mg of L-theanine.

Out of 123 individuals screened, 106 were randomized (Mg-Teadiola: *n* = 51, placebo: *n* = 55, Figure 2) and 100 completed the study (Mg-Teadiola: *n* = 49, placebo: *n* = 51). Overall, baseline characteristics (Table 1) were balanced between the two groups. Median age (interquartile range, IQR) was 25 (21, 35) years in the Mg-Teadiola group and 25 (22, 35) years in the placebo group. Proportions of men and women (women: Mg-Teadiola, *n* = 24 (49.0%); placebo, *n* = 29 (56.9%)) as well as body mass indices (median (IQR), Mg-Teadiola: 21.7 [19.8, 25.1]; placebo: 22.3 [20.7, 25.7]) were comparable in both groups.

### 3.2. Efficacy Outcomes

**Figure 3:**
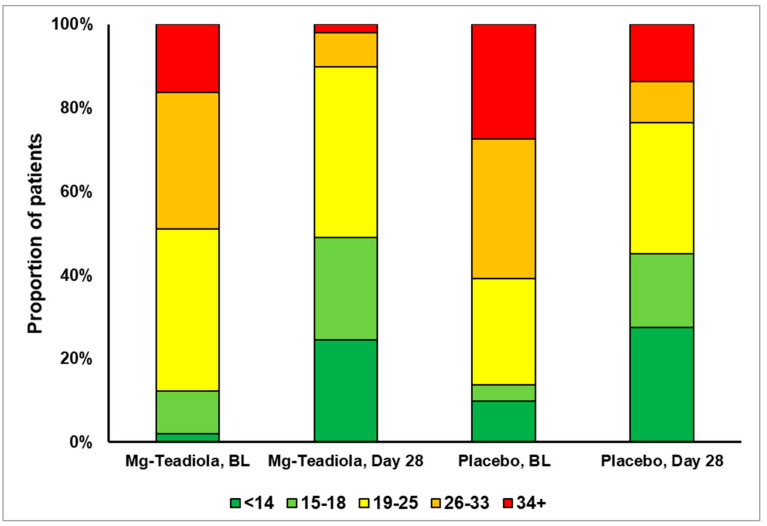
Proportion of participants with change in DASS-42 stress scores over time. BL, baseline; DASS, Depression Anxiety Stress Scale. Stress scores ranges: 0–14 (normal), 15–18 (mild), 19–25 (moderate), 26–33 (severe), and 34+ (extremely severe).

The distribution of participants showing improvement in stress levels from baseline to Day 28 is depicted in Figure 3. The proportion of participants who transitioned from extremely severe stress at baseline to mild stress on Day 28 was higher in the Mg-Teadiola group than in the placebo group (Mg-Teadiola: *n* = 8 to *n* = 1 (87.5%); placebo: *n* = 14 to *n* = 7 (50%); Figure 3). 

**Figure 4:**
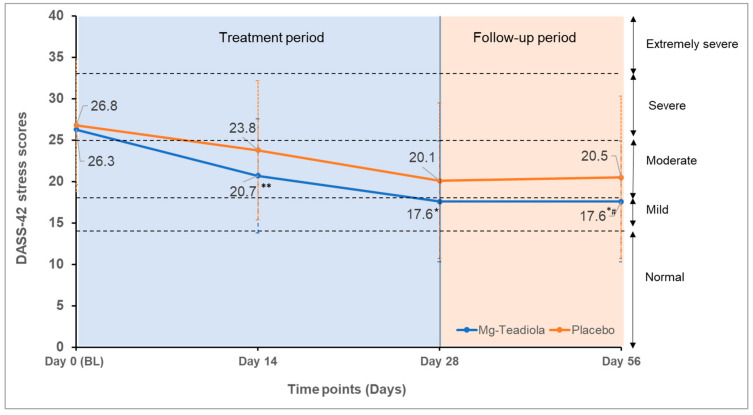
DASS-42 stress scores over time with Mg-Teadiola versus placebo. Mean (±SD) values are presented. Error bars represent SD. * *p* = 0.04 vs. Placebo; ** *p* = 0.006 vs. Placebo; * ^#^ *p* = 0.02 vs. Placebo. BL, baseline; DASS, Depression Anxiety Stress Scale; Mg-Teadiola, combination of 150 mg Mg, 0.7 mg vitamin B6, 0.1 mg vitamin B9, and 1.25 µg vitamin B12, +222 mg of rhodiola extract and 125 mg of green tea extract including 50 mg of L-theanine; SD, standard deviation. Stress scores ranges: 0–14 (normal); 15–18 (mild), 19–25 (moderate); 26–33 (severe) and 34+ (extremely severe).

The DASS-42 stress scores decreased from baseline to Day 28 in both groups (Table 1, Figure 4); however, a clinically and statistically significant decrease of three points with Mg-Teadiola versus placebo was noted (ES −0.29, 95% CI −0.57 to −0.01; *p* = 0.04), which remained stable up to Day 56 (ES, −0.34; 95% CI −0.62 to −0.06, *p* = 0.02). A similar reduction of three points with Mg-Teadiola compared with placebo was observed earlier, on Day 14 (ES, −0.40; 95% CI −0.68 to −0.11; *p* = 0.006, Table 1).

Treatment with Mg-Teadiola resulted in a significant reduction in sensitivity to cold pain compared with placebo on Day 28 (ES: −0.36, 95% CI −0.64 to −0.08; *p* = 0.01) and a trend for lower sensitivity to warm pain was observed on Day 28 (ES 0.27, 95% CI −0.01 to 0.55, *p* = 0.06, Table 1).

No significant difference was observed between the Mg-Teadiola and placebo groups with respect to the DASS-42 scores for anxiety and depression, and the PCS scores for catastrophism on Days 14, 28, and 56 (Table 1). Similarly, on Day 14, the PSQI sleep parameter scores (components 1–7) did not differ between the two groups. Interestingly, on Day 28, a trend toward an increase in the PSQI-component 7 score (daytime dysfunction due to sleepiness) with Mg-Teadiola was observed (ES −0.27, 95% CI −0.55 to 0.01; *p* = 0.06); this improvement was significant on Day 56 (ES −0.54, 95% CI −0.82 to −0.26], *p* < 0.001; Table 1).

**Figure 5:**
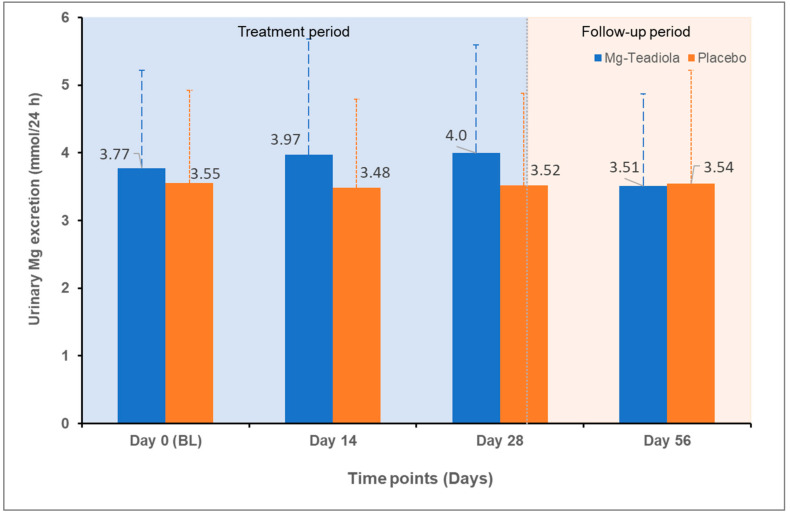
Excretion of magnesium in urine over time with Mg-Teadiola versus placebo. Values are presented as Mean (SD). Error bars represent SD. BL, baseline; Mg, magnesium; Mg-Teadiola, combination of 150 mg magnesium, 0.7 mg vitamin B6, 0.1 mg vitamin B9, and 1.25 µg vitamin B12, +222 mg of rhodiola extract and 125 mg of green tea extract including 50 mg of L-theanine; SD, standard deviation.

At baseline, the concentration of Mg in blood was 0.87 mmol/L in both groups, and overall, it remained stable on Days 14, 28, and 56. A slight increase in the level of Mg in urine from baseline to Days 14 and 28 was observed in the Mg-Teadiola group compared with the placebo group (*p* = 0.12 and *p* = 0.11, respectively; Table 1, Figure 5).

**Figure 6:**
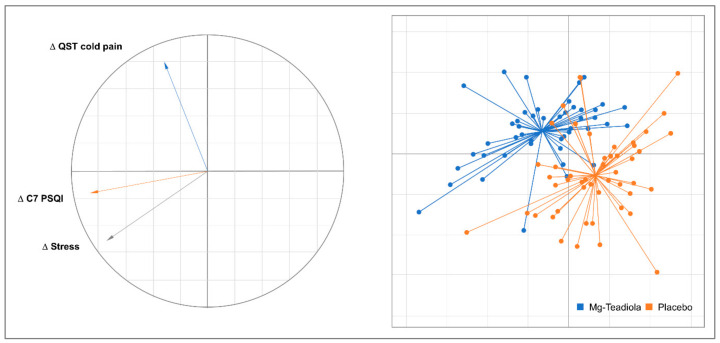
Factorial Discriminant Analyses. Δ denotes a change on Day 28 for all participants (both Mg-Teadiola and placebo groups) C7 PSQI, Component 7 (daytime dysfunction) of PSQI, Mg-Teadiola, combination of 150 mg Mg, 0.7 mg vitamin B6, 0.1 mg vitamin B9, and 1.25 µg vitamin B12, +222 mg of rhodiola extract and 125 mg of green tea extract including 50 mg of L-theanine; PSQI, Pittsburgh Sleep Questionnaire Index; QST, Quantitative Somatosensory Thermotest.

The FDA showed that Mg-Teadiola and placebo groups were discriminated for the first component of FDA (50% of the inertia) according to the changes on Day 28 for DASS stress, cold pain QST, and component 7 (daytime dysfunction) of the PSQI score (Figure 6). Interestingly, according to the second component of the FDA (35% of inertia), this discrimination seemed to distinguish participants with high values for DASS stress changes and component 7 of the PSQI score change (Figure 6, left side, bottom) from participants with high values for cold pain QST changes (Figure 6, left side, top).

### 3.3. Safety Outcomes

One serious AE (SAE) was reported in the Mg-Teadiola group; a participant tested positive for pregnancy at the end-of-study visit (Visit 5). This SAE was not considered related to the study treatment. No SAEs were reported in the placebo group. A total of 75 non-serious AEs in the Mg-Teadiola group and 91 AEs in the placebo group were reported (Supplementary Table S1). The AE with the highest frequency was headache or migraine (16/51, 31.4% versus 21/55, 38.2% participants). None of the AEs were considered treatment-related.

## 4. Discussion

This clinical study, conducted in otherwise healthy individuals with chronic stress, demonstrated the effectiveness of Mg-Teadiola compared with placebo on Day 14 (*p* = 0.006) and Day 28 (*p* = 0.04). The study also showed a trend toward improvement in the QoL in terms of improvement in daytime dysfunction due to sleepiness. Supplementation with Mg-Teadiola resulted in a reduction of sensitivity to cold pain and a trend for lower sensitivity to warm pain on Day 28. No significant effects of treatment were noted on other factors including anxiety and depression.

The DASS−42 stress scores decreased from baseline to Day 28 for both the Mg-Teadiola and placebo groups (by 33% and 25%, respectively), pointing toward a placebo effect. However, the difference in the DASS−42 stress scores was considered as clinically significant (3-point decrease), in favor of Mg-Teadiola. This effect was observed as early on as Day 14 and remained stable up to Day 56, even after discontinuation of the supplementation at Day 28. These results confirm the benefits of Mg-Teadiola on stress, in line with a previous study conducted in individuals under acute stress exposure [12]. The mechanism of action of Mg-Teadiola in stress reduction observed in this study is expected to be linked to a synergism or an additive effect of the ingredients Mg, B vitamins, rhodiola, and green tea [12].

To our knowledge, this study is the first to demonstrate a significant clinical effect of a dietary supplement on stress relief after only 14 days of treatment. We identified the few randomized clinical trials conducted with food supplements using the same methodology to evaluate stress (DASS−42 or DASS−21). A few probiotic strains such as *Lactobacillus plantarum* DR7 or P8 [34,35], and herbal extracts such as Saffron [36] or Ashwagandha [37,38] have shown potential for beneficial effects on stress or mental health, generally after 4 to 8 weeks of use. These studies have mainly included participants with lower severity of stress at baseline (mild to moderate on average), and the difference in methodology makes the comparison of efficacy between these studies difficult. However, none reported a significant effect on stress before one month of supplementation.

Both green tea (L-theanine) and rhodiola offer acute functional benefits under stress conditions [39,40], and they might have contributed to the study findings. A previous study by Boyle et al. [12] highlighted the important and synergistic role of rhodiola and green tea extracts in the products’ beneficial effects on acute stress. Green tea is comprised of flavonoids, including L-theanine, and its acute intake in the form of tea or supplements is associated with functional benefits on subjective, physiological, and psychological states in acute stress conditions [6,12,39,41]. These include enhanced subjective relaxation, calmness, mood, reduced vigilance and stress, anxiety, cortisol, and heart-rate responses [6,12,42,43,44]. Rhodiola has been used as a traditional medicine to relieve stress, anxiety, and fatigue. Preclinical and clinical data support the stress-lowering effect of rhodiola in acute and chronic stress [8,12,45,46]. In addition, its acute intake helps in building resilience and managing physical stress along with reduced cardiovascular responses [47,48].

The efficacy of Mg has been well established for stress and its comorbidities [7,49,50]. A recent study showed the potential benefit of Mg, with or without vitamin B6, on mental health and QoL [9,51]. Results showed a reduction (improvement) in the DASS−42 stress scores by 27% to 32% on Day 28 compared to baseline, in alignment to that observed in the present study (Mg-Teadiola: Day 28, 33%) [9]. Furthermore, vitamin B6 brought additional benefits to the population with a high level of stress at baseline. Interestingly, the severity of stress at baseline in the previous study was also comparable (DASS stress score of 27.7 versus 26.3–26.8 in the present study). However, the results of these studies must be compared with caution considering the differences in the inclusion criteria of the participants (e.g., magnesemia), the dose of Mg used for supplementation (300 mg vs. 150 mg in the present study), and the lack of a placebo-control arm in the previous study. Thus, Mg may be beneficial in stress reduction—especially in individuals with low magnesemia [9]. Although the participants did not appear to be deficient in Mg (showing normomagnesemia) at the start of this study, it is important to note that during the treatment period, excretion of Mg in urine tended to increase from baseline to all the visits with Mg-Teadiola compared with placebo, indicative of a saturation of Mg body stores in the Mg-Teadiola group participants. Given the relation of stress to Mg [50], this could contribute to the observed benefit of the Mg-Teadiola on stress.

Mg-Teadiola dietary supplements showed a minor effect on secondary QoL parameters, notably a reduction in the perception of cold pain. The relationship between stress and perception of pain is well known [19,52]. Stress has also been shown to be a key contributor to nociplastic pain and related disorders including fibromyalgia [53,54] or irritable bowel syndrome (IBS) [55]. Dietary interventions including Mg have demonstrated benefits on pain perception [56] and are used to manage symptoms of fibromyalgia [57]. Mg supplementation improves pain relief [58]. A recent study based on the animal model of chronic pain indicated that a single dose of a Mg salt appeared to cause a weakening in the transmission and perception of nociceptive pain [59]. At the cellular level, cold temperature detection involves the process of sensory transduction in cutaneous primary sensory nerve terminals, which converts thermal stimuli into depolarizations of the membrane. A large array of ion channels, including leak K+ and voltage-gated Na+ and K+ channels, shapes this process for the transduction of cold by nociceptors and in cold-induced pain [60]. The exact role of magnesium and other components of Mg-Teadiola in modulating cold pain occurrence at the channel and receptor level remains to be seen. Nevertheless, this finding opens avenues of research in oxaliplatin-induced cancer pain characterized by exacerbated pain to cold stimuli. Furthermore, patients with cancer also suffer from sleep disturbances and stress and have few therapeutic options to prevent and alleviate pain in this condition [61].

Adaptogens including rhodiola augment adaptation to pain stimuli [62]. The protective effect of green tea (L-theanine) on neuropathic pain has also been demonstrated in animal models [63]. Therefore, it may be hypothesized that the effect of Mg-Teadiola on pain perception observed in this study could possibly be linked to synergism or the additive effects of key ingredients in Mg-Teadiola. Taken together, the beneficial effects of Mg-Teadiola on stress may be extended to patients with pain, fibromyalgia, IBS, and other disorders triggered by stress—thus improving the overall QoL. Further research is needed to assess the role of Mg-Teadiola in managing nociplastic pain and other stress-related disorders.

No treatment-related AEs with Mg-Teadiola or placebo were identified during this study. One of the participants, who tested positive for pregnancy in the Mg-Teadiola group, chose a voluntary termination of pregnancy. This SAE had no impact on the participant’s health or the study.

A notable strength of this study is the findings noted during the follow-up period. The results observed on Day 28 for both groups were remarkably stable up to Day 56 (e.g., stress and daytime dysfunction due to sleepiness), one month after discontinuing the supplementation, reinforcing the conclusion on the potential benefits of Mg-Teadiola in the management of stress and improving QoL.

This study has a limitation: it was single-blinded (investigator-blinded) as the placebo and Mg-Teadiola tablets lacked structural similarity due to manufactural constraints. Nevertheless, measures were taken to maximize blinding of participants to the treatment; as participants were naïve to any Mg treatment, it was expected that they would not be able to recognize the treatment administered. Considering that this was a parallel trial, participants from one arm did not receive treatment from the other arm and appropriate measures were adopted so that participants did not meet throughout their participation in the study. Furthermore, gender-specific analyses and evaluation of hormonal profiles of female participants were not planned in the study protocol. However, it is worthwhile to consider these in future studies.

## 5. Conclusions

Mg-Teadiola—a combination of Mg, B vitamins, rhodiola, and green tea (L-theanine)—was effective in relieving stress on Days 14 and 28 in chronically stressed but otherwise healthy individuals. This finding, in addition to the observation that Mg-Teadiola may diminish pain perception, underlines its potential benefits for patients suffering from pain, in whom comorbidities such as stress and sleep disorders are frequent. This deserves further attention in future studies.

## Figures and Tables

**Table 1:** DASS-42 stress, PCS-catastrophism, and PSQI-sleep scores; sensitivity to pain; and magnesium levels at baseline and all visits, following supplementation with Mg-Teadiola versus placebo.

	Visit 2 (Baseline)		Visit 3 (Day 14)			Visit 4 (Day 28)			Visit 5 (Day 56)		
	Mg-Teadiola *n* = 49	Placebo *n* = 51	Mg-Teadiola *n* = 49	Placebo *n* = 51	ES (95%CI), *p*-Value	Mg-Teadiola *n* = 49	Placebo *n* = 51	ES (95%CI), *p*-Value	Mg-Teadiola *n* = 49	Placebo *n* = 51	ES (95%CI), *p*-Value
**DASS stress**	26.3 ± 6.7	26.8 ± 8.0	20.7 ± 6.9	23.8 ± 8.4	−0.40 (−0.68; −0.11), **0.006**	17.6 ± 7.3	20.1 ± 9.4	−0.29 (−0.57; −0.01), **0.04**	17.6 ± 7.3	20.5 ± 9.8	−0.34 (−0.62; −0.06], **0.02**
DASS anxiety	14.2 ± 7.8	14.1 ± 8.1	11.1 ± 6.7	10.6 ± 7.2	0.07 (−0.21; 0.35), 0.61	9.1 ± 6.8	9.2 ± 7.7	−0.02 (−0.30; 0.26), 0.90	8.5 ± 6.7	8.8 ± 7.6	−0.05 (−0.33; 0.23) 0.75
DASS depression	13.4 ± 10.2	13.4 ± 8.4	9.0 ± 8.7	8.3 ± 6.9	−0.06 (−0.34; 0.22), 0.68	7.0 ± 6.7	7.1 ± 6.3	−0.14 (−0.43; 0.13), 0.31	7.5 ± 8.4	6.8 ± 6.7	−0.05 (−0.33; 0.23), 0.70
**PCS**	16.9 ± 9.9	15.3 ± 10.9	14.5 ± 10.0	14.0 ± 10.6	−0.07 (−0.35; 0.21), 0.62	13.0 ± 10.8	12.6 ± 10.5	−0.08 (−0.36; 0.20), 0.57	12.5 ± 11.2	12.7 ± 10.6	−0.14 (−0.42; 0.14), 0.34
Rumination	6.0 ± 4.1	5.6 ± 4.6	5.2 ± 4.1	4.9 ± 4.1	0.02 (−0.26; 0.30), 0.90	4.3 ± 3.8	4.5 ± 4.3	−0.08 (−0.36; 0.20), 0.57	3.8 ± 3.9	4.2 ± 3.8	−0.15 (−0.43; 0.13), 0.30
Magnification	4.6 ± 2.7	4.1 ± 2.9	3.6 ± 2.7	3.8 ± 2.9	−0.19 (−0.47; 0.09), 0.19	3.4 ± 3.0	3.4 ± 2.6	−0.10 (−0.38; 0.18), 0.49	3.4 ± 3.1	3.5 ± 2.8	−0.11 (−0.39; 0.17), 0.44
Helplessness	6.3 ± 4.7	5.6 ± 5.0	5.7 ± 4.3	5.3 ± 5.1	−0.02 (−0.30; 0.26), 0.87	5.3 ± 4.9	4.8 ± 5.1	0.00 (−0.28; 0.28), 0.99	5.3 ± 5.1	5.1 ± 5.1	−0.07 (−0.35; 0.21), 0.61
**PSQI-sleep**	7.2 ± 2.5	7.8 ± 2.9	6.3 ± 2.4	7.0 ± 3.1	−0.14 (−0.42; 0.14), 0.33	5.6 ± 2.6	6.2 ± 3.1	−0.10 (−0.38; 0.18), 0.49	5.3 ± 2.4	3.2 ± 3.1	−0.07 (−0.35; 0.21), 0.63
Component 1	1.6 ± 0.7	1.6 ± 0.7	1.4 ± 0.7	1.5 ± 0.8	−0.07 (−0.35; 0.21), 0.64	1.3 ± 0.7	1.4 ± 0.7	−0.02 (−0.30; 0.26), 0.90	1.3 ± 0.6	1.3 ± 0.7	−0.01 (−0.28; 0.28), 0.99
Component 2	1.8 ± 1.0	1.8 ± 1.0	1.4 ± 1.0	1.3 ± 1.1	−0.14 (−0.42; 0.14), 0.33	1.2 ± 1.0	1.4 ± 1.1	−0.15 (−0.43; 0.14), 0.31	1.4 ± 0.9	1.5 ± 1.0	−0.09 (−0.37; 0.20), 0.55
Component 3	0.8 ± 0.7	0.9 ± 0.8	0.7 ± 0.7	0.8 ± 0.8	−0.05 (−0.33; 0.23), 0.74	0.7 ± 0.8	0.6 ± 0.7	0.21 (−0.07; 0.49), 0.14	0.7 ± 0.7	0.6 ± 0.7	0.15 (−0.13; 0.43), 0.30
Component 4	0.3 ± 0.6	0.6 ± 0.8	0.3 ± 0.5	0.5 ± 0.9	−0.20 (−0.48; 0.08), 0.17	0.3 ± 0.6	0.4 ± 0.7	−0.10 (−0.38; 0.18), 0.50	0.3 ± 0.5	0.4 ± 0.8	0.05 (−0.23; 0.33), 0.72
Component 5	1.3 ± 0.5	1.4 ± 0.5	1.3 ± 0.4	1.3 ± 0.4	0.07 (−0.21; 0.35), 0.63	1.2 ± 0.4	1.2 ± 0.5	−0.02 (−0.30; 0.26), 0.88	1.2 ± 0.4	1.1 ± 0.5	0.22 (−0.06; 0.50), 0.13
Component 6	0.1 ± 0.2	0.2 ± 0.6	0.1 ± 0.1	0.2 ± 0.6	−0.17 (−0.45; 0.11), 0.23	0.1 ± 0.1	0.1 ± 0.5	−0.12 (−0.40; 0.16), 0.39	0.1 ± 0.3	0.2 ± 0.6	−0.02 (−0.30; 0.26), 0.88
Component 7	1.4 ± 0.6	1.4 ± 0.7	1.2 ± 0.6	1.2 ± 0.7	−0.03 (−0.31; 0.25), 0.85	0.8 ± 0.7	1.1 ± 0.8	−0.27 (−0.55; 0.01), 0.06	0.7 ± 0.6	1.2 ± 0.8	−0.54 (−0.82; −0.26), **<0.001**
**Pain, QST,** **°C**			Data not collected						Data not collected		
Warm sensibility	34.0 ± 1.3	33.5 ± 0.7				33.8 ± 0.8	33.9 ± 1.2	−0.07 (−0.35; 0.21), 0.61			
Cold sensibility	30.8 ± 1.1	31.0 ± 0.7				30.7 ± 0.9	30.6 ± 1.2	0.17 (−0.11, 0.45), 0.22			
Warm pain	42.5 ± 3.7	42.5 ± 3.5				43.2 ± 3.2	42.2 ± 2.9	0.27 (−0.01; 0.55), 0.06			
Cold pain	17.3 ± 10.7	18.0 ± 9.9				15.6 ± 10.3	19.9 ± 9.3	−0.36 (−0.64; −0.08), **0.01**			
**Concentration of Mg**											
Plasma, mmol/L	0.87 ± 0.07	0.87 ± 0.06	0.87 ± 0.06	0.87 ± 0.06	0.08 (−0.33; 0.48), 0.71	0.87 ± 0.01	0.88 ± 0.01	−0.26 (−0.14; 0.66), 0.20	0.88 ± 0.06	0.88 ± 0.05	−0.02 (−0.41; 0.38), 0.94
Urine, mmol/24 h	3.77 ± 1.45	3.55 ± 1.37	3.97 ± 1.71	3.48 ± 1.31	0.32 (−0.08; 0.72), 0.12	4.00 ± 1.59	3.52 ± 1.36	0.32 (−0.07; 0.72), 0.11	3.51 ± 1.36	3.54 ± 1.68	0.32 (−0.08; 0.72), 0.92

Values are mean+/− SD unless otherwise stated. Significant *p*-values are in bold. PSQI component 1: subjective sleep quality; PSQI component 2: sleep latency; PSQI component 3: sleep duration; PSQI component 4: habitual sleep efficiency; PSQI component 5: sleep disturbances; PSQI component 6: use of sleeping medication; PSQI component 7: daytime dysfunction; QST, Quantitative Somatosensory Thermotest. DASS, Depression Anxiety Stress Scale; ES, effect size; Mg, magnesium; Mg-Teadiola, combination of 150 mg Mg, 0.7 mg vitamin B6, 0.1 mg vitamin B9, and 1.25 µg vitamin B12, + 222 mg of rhodiola extract and 125 mg of green tea extract including 50 mg of L-theanine; PCS, Pain Catastrophizing Scale; PSQI, Pittsburgh Sleep Questionnaire Index.

## Data Availability

Due to the proprietary nature of the study, participant data are not available in public domain.

## Supplementary Materials

The following supporting information can be downloaded at: https://www.mdpi.com/article/10.3390/nu14091863/s1, File S1: Exclusion criteria; Table S1: Non-serious adverse events reported in the study (safety set).
