# Supplementary material for: Effect of a Combination of Magnesium, B Vitamins, Rhodiola, and Green Tea (L-Theanine) on Chronically Stressed Healthy Individuals—A Randomized, Placebo-Controlled Study
Source: Nutrients. 2022 Apr 29;14(9):1863. doi: 10.3390/nu14091863 (PMC9102162; doi:10.3390/nu14091863)
Supplement: Supplementary file 1 [file nutrients-14-01863-s001.zip › File S1.pdf]

Supplementary material

Exclusion criteria

- Contraindication to Mg administration: hypersensitivity to magnesium chloride, or to any of the excipients
- Magnesium content >1.05 mmol/L
- Severe renal failure with creatinine clearance <60 mL/min
- Receiving a treatment or food supplement containing Mg at the time of inclusion,
- Treated with antibiotics during the 3 months prior to inclusion
- Medical and/or surgical history considered by the investigator or his representative to be incompatible with the trial (in particular a disease known to cause neuropathies of small fibers: diabetes, Gougerot-Sjögren's disease, vasculitis, sarcoidosis, chronic ethylism, etc.)
- Medical and/or surgical history deemed by the investigator or his representative to be incompatible with the trial
- Evolutionary pathology at the time of the inclusion assessment
- Excessive consumption of alcohol, tobacco (+10 cigarettes/day), coffee, tea or drinks containing caffeine (equivalent to more than 4 cups/day) or drug abuse
- Patient who did not meet the selection criteria regarding their ability to discriminate against sensations resulting from nociceptive stimulation during psychometric tests
- Individuals who did not meet the selection criteria regarding their ability to discriminate against projected colours during the fMRI examination
- Individuals participating in another clinical trial, or in the exclusion period, or received a total amount of compensation exceeding 4500 Euros over the 12 months preceding the start of the trial
- A woman of childbearing age who was not using an effective contraceptive method, pregnant, or breastfeeding
