# Supplementary material for: Effect of a Combination of Magnesium, B Vitamins, Rhodiola, and Green Tea (L-Theanine) on Chronically Stressed Healthy Individuals—A Randomized, Placebo-Controlled Study
Source: Nutrients. 2022 Apr 29;14(9):1863. doi: 10.3390/nu14091863 (PMC9102162; doi:10.3390/nu14091863)
Supplement: Supplementary file 1 [file nutrients-14-01863-s001.zip › Table S1.pdf]

Supplementary material

Table S1. Non-serious adverse events reported in the study (safety set).

**AE (verbatim)**	**Mg-Teadiola n=51**		**Placebo n=55**	
	**Events n**	**Number of participants n′ (%)**	**Events n**	**Number of participants n′ (%)**
Headaches/migraines	23	16 (31.4)	32	21 (38.2)
Musculoskeletal pain	14	12 (23.5)	11	9 (16.4)
Dental pain	1	1 (2.0)	5	3 (5.5)
Fatigue	6	4 (7.8)	3	3 (5.5)
Gynecological disorders	0	0 (0.0)	13	8 (14.5)
Digestive disorders	16	10 (19.6)	12	10 (18.2)
Dermatological disorders	3	3 (5.9)	3	3 (5.5)
Infections COVID	2	2 (3.9)	1	1 (1.8)
Urinary tract infections	0	0 (0.0)	2	2 (3.6)

AE, adverse event; COVID, coronavirus disease; n, number of AEs, n′, number of participants.
